# Seagrass Restoration Enhances “Blue Carbon” Sequestration in Coastal Waters

**DOI:** 10.1371/journal.pone.0072469

**Published:** 2013-08-14

**Authors:** Jill T. Greiner, Karen J. McGlathery, John Gunnell, Brent A. McKee

**Affiliations:** 1 Department of Environmental Sciences, University of Virginia, Charlottesville, Virginia, United States of America; 2 Department of Marine Sciences, University of North Carolina, Chapel Hill, Chapel Hill, North Carolina, United States of America; MESC; University of South Alabama, United States of America

## Abstract

Seagrass meadows are highly productive habitats that provide important ecosystem services in the coastal zone, including carbon and nutrient sequestration. Organic carbon in seagrass sediment, known as “blue carbon,” accumulates from both *in situ* production and sedimentation of particulate carbon from the water column. Using a large-scale restoration (>1700 ha) in the Virginia coastal bays as a model system, we evaluated the role of seagrass, *Zostera* *marina* , restoration in carbon storage in sediments of shallow coastal ecosystems. Sediments of replicate seagrass meadows representing different age treatments (as time since seeding: 0, 4, and 10 years), were analyzed for % carbon, % nitrogen, bulk density, organic matter content, and ^210^Pb for dating at 1-cm increments to a depth of 10 cm. Sediment nutrient and organic content, and carbon accumulation rates were higher in 10-year seagrass meadows relative to 4-year and bare sediment. These differences were consistent with higher shoot density in the older meadow. Carbon accumulation rates determined for the 10-year restored seagrass meadows were 36.68 g C m^-2^ yr^-1^. Within 12 years of seeding, the restored seagrass meadows are expected to accumulate carbon at a rate that is comparable to measured ranges in natural seagrass meadows. This the first study to provide evidence of the potential of seagrass habitat restoration to enhance carbon sequestration in the coastal zone.

## Introduction

Seagrass meadows are essential coastal ecosystems that provide many ecosystem services such as improved water quality and light availability, increases in biodiversity and habitat, sediment stabilization, and carbon and nutrient accumulation [1–3]. Recently, seagrass meadows have been acknowledged for their carbon storage potential and it has been estimated that globally as much as 19.9 Pg of organic carbon are stored in seagrass meadows [4]. Seagrass meadows cover only 0.1% area of the world’s ocean floor, yet account for 10–18% of the total oceanic carbon burial, accumulating carbon at rates of 48 to 112 Tg C yr^-1^ [5–7]. Globally, seagrass ecosystems are declining in area by about 5% per year due to anthropogenic stresses, including decreased water quality and increased water temperatures [2,7,8], and this decline could result in the release of large amounts of stored carbon [5]. In order to partially mitigate seagrass decline, restoration in areas with suitable habitat is an effective option that has the potential to reestablish lost carbon stores and sinks, as well as other important ecosystem services seagrass meadows provide.

Carbon accumulation in marine sediments provides long-term storage of organic carbon and has been referred to as “blue carbon” to distinguish it from carbon in terrestrial sinks [9]. Unlike terrestrial systems that store organic carbon primarily in living biomass and soil organic matter, coastal vegetated systems store the majority of organic carbon in sediment [4,5,7]. In addition, terrestrial habitats lose carbon stocks to the atmosphere as CO_2_ via decomposition or by disturbances such as fires [10,11]. Because marine sediments are often anoxic and continually accumulate sediment [6], organic carbon can be preserved within them over decadal to even millennial time scales where the organic carbon, though subjected to some diagenesis, is still considered a carbon sink [12–14]. Fast accumulation rates, low oxygen, low sediment hydraulic conductivity, and slower microbial decomposition rates facilitate carbon burial and the accumulation of carbon stocks in these coastal sediments [11,15,16].

The dense canopy of seagrass meadows reduces flow velocity [17,18], which subsequently promotes the deposition of sediment and particles from the water column [19–21] and reduces sediment resuspension [19]. When sediments of seagrass meadows are compared to unvegetated sediments, there can be as much as a threefold reduction in resuspension of fine-grained sediment [17–19]. The particles that are trapped and deposited in seagrass-vegetated sediments are often rich in organic matter (OM), averaging 4.1% [6]. However, this trapping effect is reduced with decreased seagrass density, which could be driven by natural and human stresses on seagrass meadows such as storm disturbance or eutrophication [1,19].

Carbon accumulation rates for established seagrass meadows vary depending on the seagrass species, sediment characteristics, and depth range of the seagrass habitats. From a global survey of 123 sites, the average carbon burial rate was 138 ± 38 g C m^-2^ yr^-1^ (mean ± SE, range = 45–190); the large range in rates reflects variation in shallow and deep habitats for both tropical and temperate seagrass [7]. Currently, there are only a few measurements of carbon accumulation from different seagrass species such as *Posidonia* *oceanica* , *Cymodocea* *nodosa* , and *Zostera* *marina* , and no carbon accumulation measurements in restored seagrass meadows [5,7,11]. Many studies lack accurate estimates of carbon burial due to the absence of direct measurements of key variables such as sediment bulk density and sedimentation rates [4]. In addition, many studies do not specify whether roots are included in measurements of sediment carbon, which potentially results in variable estimates of carbon burial. Kennedy et al. [6] estimated that seagrass carbon contributed about 50% to the sediment organic carbon pool in seagrass-vegetated sediments globally, but did not distinguish between root, rhizome, and leaf material. The relative contribution of root and rhizome material is also influenced by decomposition rate, which varies among species [4]. Clarifying whether measurements include roots or not is important when determining carbon stock or accumulation measurements in seagrass meadows [11].

If the current rates of seagrass habitat decline remain unchanged, annual loss in seagrass habitat could result in the release of previously stored carbon of up to 299 Tg C yr^-1^ [4]. Seagrass habitat loss due to land-use change, based on the annual loss rate of 0.4–2.6% seagrass habitat globally, was estimated to release between 0.05 to 0.33 Pg CO_2_ yr^-1^ back into the atmosphere; this rate is comparable to the annual rates of fossil fuel CO_2_ emissions in many small countries [22]. This large evasion of CO_2_ to the atmosphere could result in an economic cost of $1.9 -13.7 billion yr^-1^ [22]. Restoration is one way to mitigate the continual loss of seagrass habitat and to prevent seagrass from becoming a significant carbon source.

Despite the recent recognition that seagrass meadows are important marine carbon stores, the potential of habitat restoration in increasing carbon stocks and sinks in coastal waters is unknown. The goal of this study was to assess carbon stores and the rate of carbon accumulation in a large-scale restoration in the Virginia coastal bays as a model system where eelgrass, *Zostera* *marina* , was broadcast seeded at several different times. Restored seagrass meadows (4 and 10 years old) and unvegetated areas were studied to determine how carbon accumulation was promoted by seagrass of varying ages.

## Methods

This study was conducted at the Virginia Coast Reserve Long Term Ecological Research site (VCR LTER) on the Eastern Shore of Virginia, on the ocean side of the Delmarva Peninsula. Prior to 1933, dense meadows of *Zostera* *marina* (eelgrass) carpeted the seafloor [23] and supported a lucrative scallop fishery. The slime-mold (*Labarinthula* sp.) wasting disease and a severe hurricane in 1933 caused a local extinction of the seagrass [23–25]. The lost seagrass habitat in the Virginia coastal bays resulted in both a decrease in sediment stabilization and a faunal reduction which resulted, notably, in the collapse of the local scallop fishing industry [2,23]. Starting in 2001, eelgrass was seeded over multiple years in South Bay and Hog Island Bay, creating a system of seagrass meadows of varying ages that comprised over 1700 ha in 2011 [3,26].

Two adjacent locations within the VCR LTER were used for this study, Hog Island Bay (HI) (37°24’47″ N, 75°43’36″ W) and South Bay (SB) (37°, 15’ 54″ N, 75°48’50″ W), both with a tidal range of approximately 1.2 m [27]. All necessary permits were obtained for field methods conducted in this study. The seagrass restoration area was set aside for restoration and seagrass research by the Virginia Marine Resource Commission; through collaboration with the Virginia Institute of Marine Sciences, permission was given to use these sites in the VCR LTER for research purposes. Previous research has determined that these two locations are similar in terms of bathymetry, water depth, sediment, water-column characteristics, and current speeds [3]. In South Bay (SB) replicate 0.4-ha plots were seeded with 100,000 seeds ha^-1^ in 2001 and in Hog Island Bay (HI) replicate 0.2- and 0.4-ha plots were seeded with 100,000 seeds ha^-1^ in 2007. These sites were used as a 10-year age treatment (SB) and a 4-year age treatment (HI), respectively. Previous analysis and monitoring found that there were no significant differences in sediment and plant properties as a result of different plot sizes or initial seed density, allowing for sites of different plot size (0.2- and 0.4-ha) to be pooled [3]. Annual monitoring showed that seagrass shoot densities increased with time since seeding, with an initial 4-year lag followed by a rapid linear increase in shoot density as a function of age [3]. In addition, surrounding unvegetated sediment was sampled at both sites (SB and HI) to represent the 0-year age, or un-restored, reference treatment. For each age treatment, 6 plots were selected for sediment core sampling, except in the 0-year (SB) treatment, where 4 plots were sampled.

Sampling was conducted during the summer of 2011 from June through August. Additional cores were taken in October 2011 at the 0-year (SB) plots. Depth profiles of sediment characteristics were not likely to be different between June through October in the unvegetated plots because these represented sediment accumulated over annual to decadal time scales. In the 10-year and 4-year seagrass plots, a 50-m (for 0.4-ha plots) and 25-m (for 0.2-ha plots) transect was placed parallel to the current and aligned with the center of each plot. Four 20-cm deep, 10-cm diameter cores were taken equidistant along each transect and processed the same day. Large cores had sharp edges and allowed water to escape when collecting to prevent compression and build up of pressure. After collection, the corer created a seal to keep the core in place during extraction from the sediment. Seagrass densities were measured by counting shoots in ten 0.25-m^2^ quadrats at regular intervals along each transect, resulting in 60 measurements per treatment.

Extruded cores were divided into 1.0-cm intervals; shells, rocks, and large rhizomes were removed and then the wet weight of each core interval was measured. Subsamples from each interval were taken to measure water content of the sediment, loss on ignition (% OM), percent carbon (% C) and percent nitrogen (% N). Dry bulk density (BD) was determined using the mass of the dry sediment 1.0-cm section, which was determined from the percent water content of the total wet weight, divided by the volume of the sediment section. Sediments were dried at 60° C for 48 h and then placed in a muffle furnace at 500° C for 6 h to determine % OM using the loss on ignition method. A portion of the subsamples was dried at 60° C for 48 h, shells were removed, the sediment homogenized by grinding, and % C and % N was measured with a Carlo Erba NA 2500 Elemental Analyzer using a helium gas carrier in a 1020° C combustion tube and 650° C reduction tube. A small subset of 24 samples was acidified with 5 N HCl before analysis of % C and % N to remove inorganic carbon. The subset was compared with non-acidified samples using a T-test.

One core from each age treatment was used to establish ^210^Pb profiles to determine sediment accretion rates. ^210^Pb content was analyzed using isotope-dilution alpha spectrometry for the ^210^Pb granddaughter isotope polonium (^210^Po), as these radioisotopes are in secular equilibrium [28]. Because the seagrass restoration occurred in the past 10 years, the half-life of ^210^Pb radio isotope was the appropriate dating method for the sediment [28]. A ^209^Po spike was added to each sample and digested in a microwave with concentrated nitric acid. Then, hydrogen peroxide and heat were used to digest the solution and extract the tracer from organic compounds [29]. Polonium was spontaneously electroplated onto stainless steel planchets and ^209^Po/^210^Po activities were measured via alpha spectrometry using silicon surface barrier detectors linked to a multi-channel analyzer [30]. To determine sediment accretion rates, a constant rate of tracer supply model (CRS) of non-steady state sediment accretion was applied to the derived excess ^210^Pb values as they changed versus mass-depth [31,32]. Sediment accretion rates were taken for each 1-cm interval using the specific date, and carbon accumulation rates were calculated by multiplying carbon density and sediment accretion rate.

For carbon, nitrogen, organic matter content, and bulk density measurements, data from each variable were averaged from the top 10 cm of each age treatment (0-year (SB) *n* = 80, 0-year (HI) *n* = 230, 4- and 10-year *n* = 240). The top 10 cm of sediment was used for analysis because the ^210^Pb profile results did not show sediment accumulation below 10 cm depth and was estimated to be older than 100 years indicating the sediment at this depth was most likely not influenced by the restoration. Additionally, the top 10 cm of sediment was within the depth range influenced by root growth. Depth profiles of % C, % N, and % OM content were determined from averages of the 1-cm intervals from the replicate cores in each age treatment.

Significant differences between age treatments in % C, % N, % OM, and BD were analyzed with a 2-way nested analysis of variance (ANOVA) to determine within group variance among each treatment and variance among the different treatments using SAS software (Version 9.2 of the SAS Systems for Windows, 2008, SAS Institute Inc.). Post hoc Ryan’s Q tests were used to determine significant differences between each treatment. Seagrass densities were analyzed with an ANOVA using SAS software to determine differences in seagrass density between the 10- and 4-year treatments.

## Results

**Figure 1:**
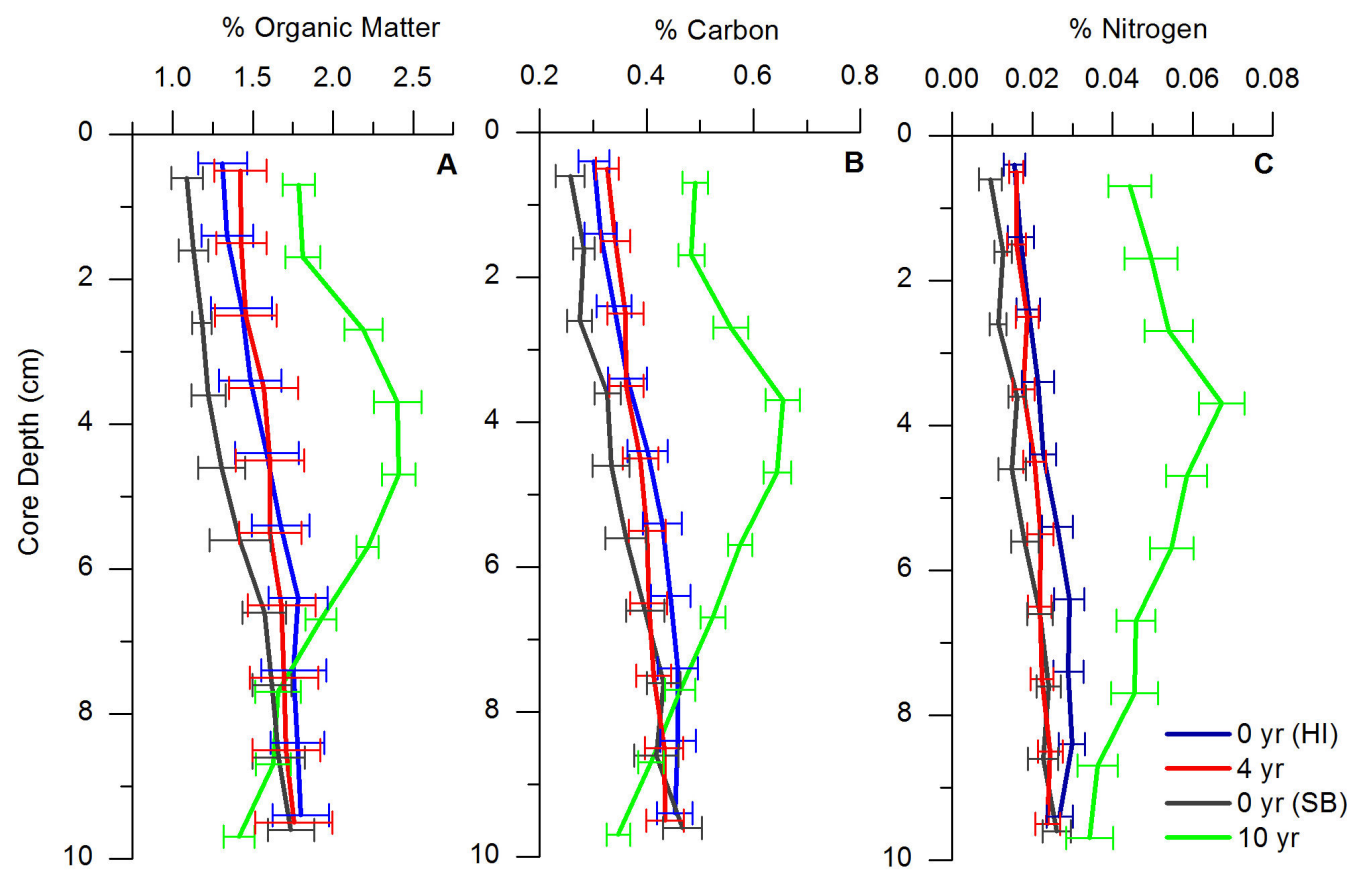
Vertical average down-core profiles of sediment characteristics in the top 10 cm. **A**) Percent organic matter (% OM); **B**) Percent organic carbon (% C); **C**) Percent nitrogen (% N) for 4 age treatments (0- (HI), 4-, 0- (SB), and 10-year) in top 10 cm of sediment, where error bars indicate standard error. Averages for each variable were calculated in 1-cm intervals to a depth of 10 cm.

Seagrass shoot densities increased significantly with bed age, where 4-year treatments averaged to 123.2 shoots m^-2^ and 10-year treatment averaged to 428.7 shoots m^-2^ (F_60,60_ = 135.89; *p* < 0.0001) (Table 1). % OM profiles of sediment cores indicated that the 10-year treatment had significantly different % OM only in the top 6 cm of sediment compared to all other age treatments, with a large increase in % OM concentrations from 3- to 6-cm depths (2.19 to 2.41% OM) (Figure 1A). There was no significant difference in average % OM between 0-year (HI) and 4-year treatments, but there was a significant difference in average % OM between 0-year (SB) and 10-year treatments (F_230,240,80,240_ = 35.20; *p* < 0.0001) (Table 1). Bulk density (BD) of sediment cores decreased significantly with age treatment, with 0-year (SB) treatment at 1.61 g cm^-3^ having the highest density compared to the 10-year treatment at 1.30 g cm^-3^ (F_240,80,240,230_ = 60.59; *p* < 0.0001) (Table 1).

**Table 1:** Mean and standard error (SE) for 4 different treatments (0-yr (HI), HI, 0-yr (SB), and SB) in the top 10 cm of sediment cores for percent carbon (% C), percent nitrogen (% N), percent organic matter (% OM), and bulk density from *n* number of samples.

**Site**	**Age** (yr)	**Seagrass Density** (Shoots m^-2^)		**% Carbon**		**% Nitrogen**		**% Organic Matter**		**Bulk Density** (g cm^-3^)		***n***
		Mean	SE	Mean	SE	Mean	SE	Mean	SE	Mean	SE	
0-yr (HI)	0	0	0	0.40	0.011	0.02	0.001	1.61	0.03	1.53	0.02	230
HI	4	123.2	39.63	0.39	0.010	0.02	0.001	1.59	0.03	1.44	0.01	240
0-yr (SB)	0	0	0	0.36	0.012	0.02	0.001	1.39	0.04	1.61	0.03	80
SB	10	428.7	30.19	0.52	0.010	0.05	0.002	1.94	0.03	1.30	0.01	240

Age indicates the number of years since seagrass was seeded, and seagrass density is the number of shoots in a square meter. 10-year (SB) was significantly different from all other treatments for all measured variables (*p* < 0.0001).

The carbon content of each age treatment varied throughout the core. Average carbon concentration in the 10-year treatment was significantly higher than the neighboring 0-year (SB) sediment and treatments in Hog Island, and with a large increase in % C in between 3- and 6-cm depths (F_240,80,240,230_ = 37.47; *p* < 0.0001) (Table 1, Figure 1B). There was no significant difference in % C between the 4-year treatment and the neighboring 0-year (HI) sediment (Table 1, Figure 1B). The % C values were taken to represent organic carbon because there was no significant difference between acidified and non-acidified sediment samples (*p* > 0.05). In addition, there was a strong linear relationship between % OM to % C for the average values from the depth intervals in the top 10-cm of sediment of each site (R^2^ = 0.96, *p* < 0.0001). Though occurring at very low concentrations, nitrogen depth profiles showed similar patterns to both % OM and % C profiles. Nitrogen concentrations were significantly higher in the 10-year treatments averaging 0.05% N, and were highest between 3- and 6-cm depths (F_240,80,240,230_ = 108.63; *p* < 0.0001) (Table 1, Figure 1C). For the other age treatments (0 and 4 years) there were no significant differences throughout the core, all with average concentration of 0.02% N (Table 1, Figure 1C).

**Figure 2:**
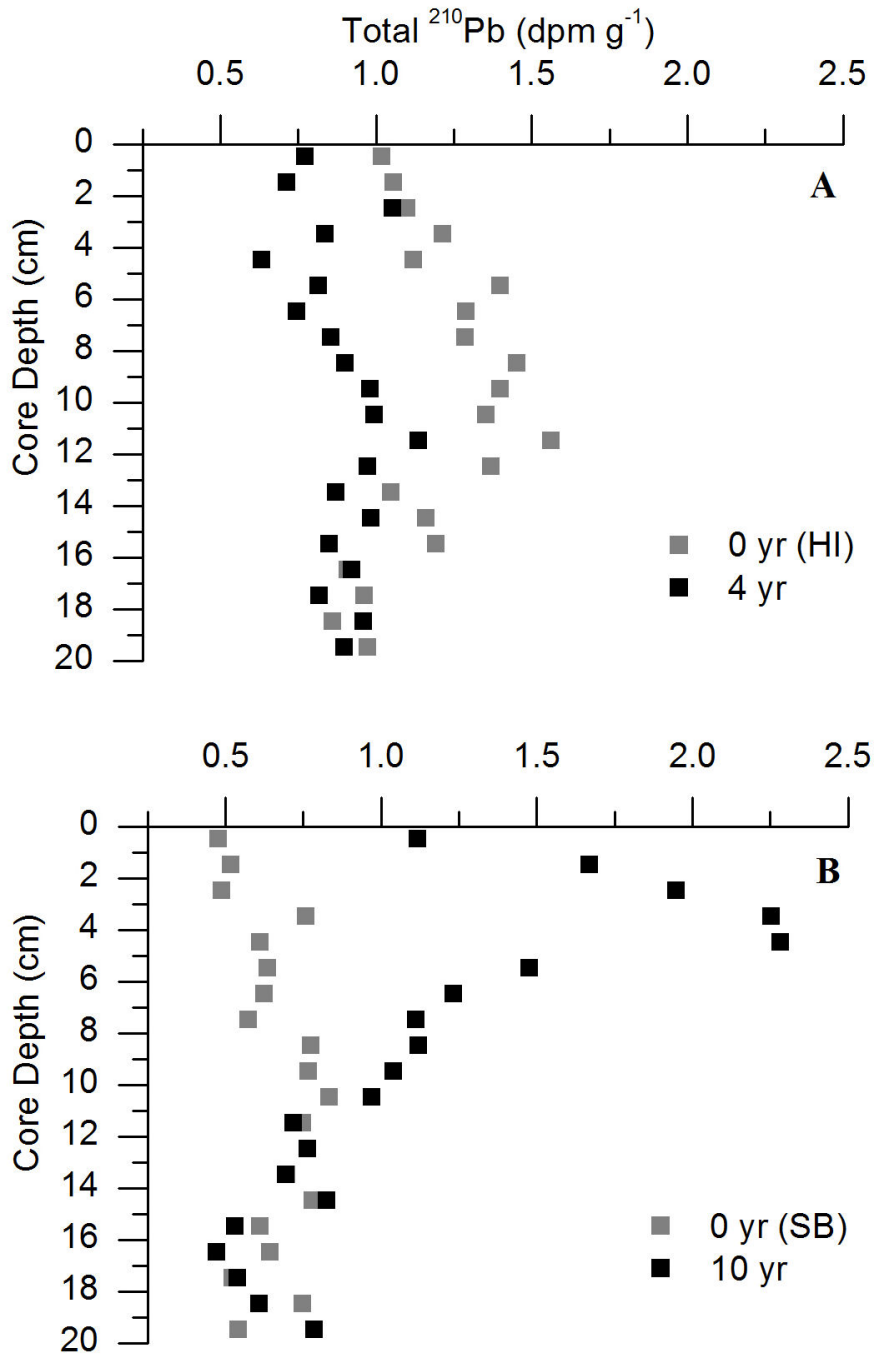
Total down core ^210^Pb activity for all treatments. **A**) Total down core ^210^Pb activity in Hog Island treatments, 0-year (HI) and 4-year. There was no significant ^210^Pb activity to determine sedimentation rate throughout the core. **B**) Total down core ^210^Pb activity in South Bay treatments, 0-year (SB) and 10-year. There was significant ^210^Pb activity in the 10-year treatment above 10 cm depth, allowing for the determination of a sedimentation rate. Error in each measurement was not significant due to low instrumental error.

**Figure 3:**
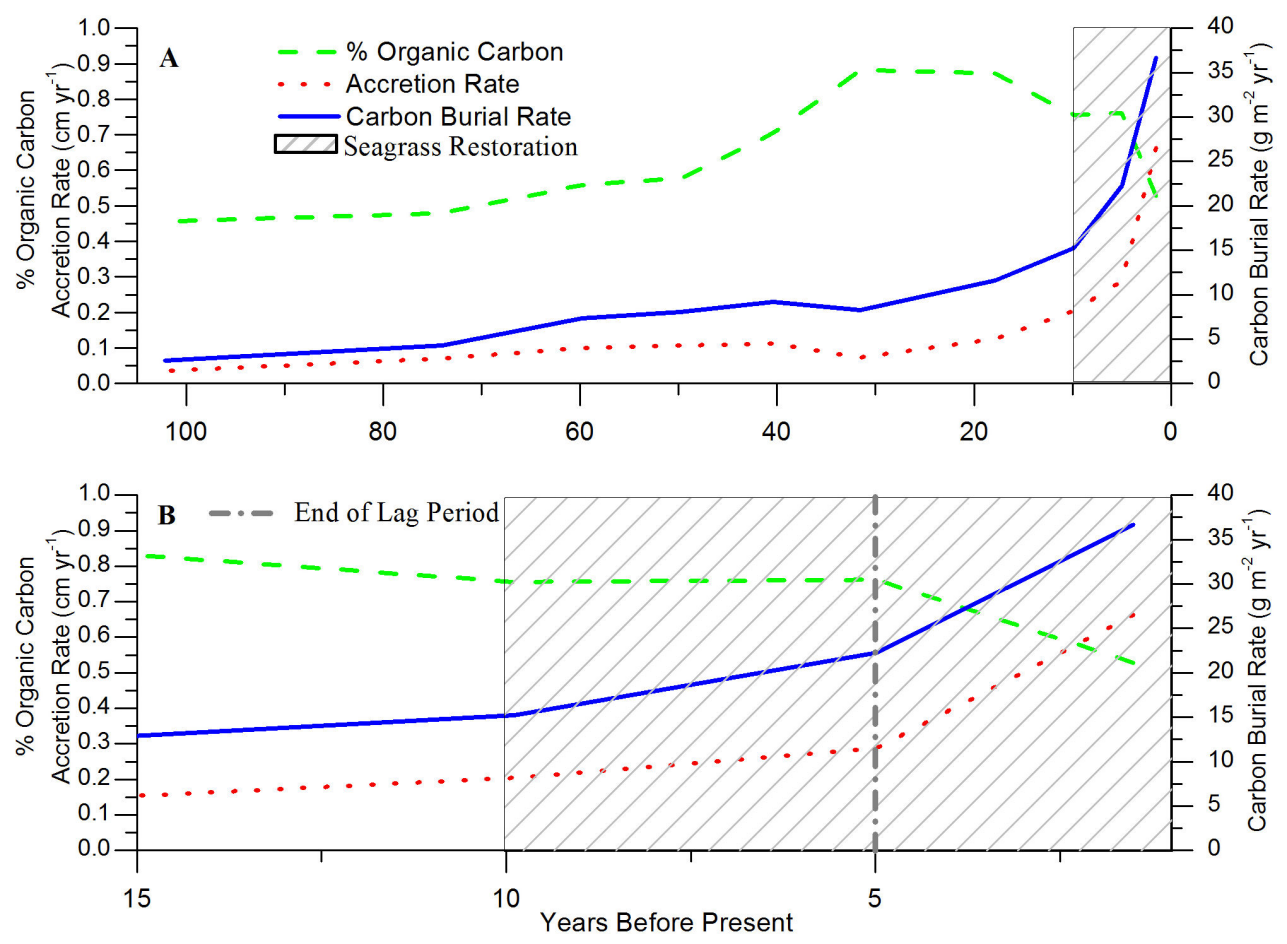
Record of sediment accretion rate, percent organic carbon, and carbon burial rate in 10-year treatment. **A**) Historical record in the 10-year treatment (SB) of sediment accretion rate, percent organic carbon, and carbon burial rate with years before present starting in 2011 (= 0 on x-axis). **B**) Recent record in the 10-year treatment of sediment accretion rate, percent organic carbon, and carbon burial rate with years before present starting in 2011. Time influenced by seagrass restoration (10 years) is enclosed in box with grey diagonal lines. The vertical, grey hyphenated line at 5 years before present indicates the end of the 5-year lag period, where before there was little change in carbon burial rates due to low seagrass density.

The vertical profile of the 10-year treatment had higher levels of excess ^210^Pb compared to the 0-year (SB) treatment (Figure 2B). ^210^Pb profiles from the 10-year and neighboring 0-year (SB) treatments conveyed a background ^210^Pb value below 10 cm depth where both the 0- and 10-year treatment values were the same, representing sediment before restoration (Figure 2B). Above 10 cm depth in the 10-year treatment, there was an excess in ^210^Pb. This indicated some accumulation of sediment over time allowing for a sediment accretion and carbon accumulation rate to be calculated as a result of the seagrass restoration (Figure 2B) [31]. However, vertical core profiles showed low and background supported ^210^Pb activity in the 4-year and 0-year treatments (Figure 2A). From the 10-year ^210^Pb profiles, sediment accretion rates were approximately 0.66 cm yr^-1^ (Figure 3). Carbon accumulation rates increased over time following the seeding, with a rapid acceleration in accretion rates starting 5 years following the seeding as the seagrass density increased. For the 10-year treatment, the seagrass accumulated approximately 36.68 (± 2.79) g C m^-2^ yr^-1^ (Figure 3).

## Discussion

Based on previous research at this site, restored seagrass meadows, measured 9 years after seeding, had three times more carbon and four times more nitrogen in the sediment; additionally, they had accumulated finer particles in the top 5 cm of sediments as compared to bare, unvegetated areas [3]. In this study, we built on these standing stock measurements to quantify, for the first time, carbon accumulation rates in restored seagrass meadows and provide evidence for the potential of seagrass habitat restoration to enhance carbon sequestration in the coastal zone.

Radioactive ^210^Pb sediment dating showed a clear pattern in the 10-year seagrass site, providing the first measurement of a restored seagrass sediment accumulation rate. The non-steady state model used to calculate the sediment accumulation rate indicated the effect of seagrass restoration. ^210^Pb profiles for the 10-year treatments showed patterns of: sediment influenced by seagrass restoration in the top few cm, sediment representing a 100 year record above 10 cm depth, and sediment unaffected by restoration or accretion below 10 cm depth (Figure 2B). The more common approach using a constant rate of supply (CRS) model to calculate sediment accumulation rate is only applicable when the flux of excess ^210^Pb into the sediment is constant over time and would be seen as a linear trend in the ^210^Pb profile. The lack of linear trend at the sediment surface of the 10-year ^210^Pb profile indicated a changing accumulation rate as the surface ^210^Pb deceased in concentration. The ^210^Pb profiles from the 4-year and neighboring 0-year (HI) treatment had values of low activity representing only the background supported ^210^Pb, indicating an insignificant impact on sediment accumulation during the first 4 years of meadow development (Figure 2A). This profile would only be seen if there was sediment older than 100 years or more likely, if the sediment profile was compromised mainly from resuspension and/or bioturbation that can cause shallow mixing creating a dilution effect. The low ^210^Pb values were not the result of deep mixing because the carbon profiles for these sites were not homogenous; as a result, sedimentation and subsequent carbon accumulation rates for the 4-year treatment could not be determined [3].

Increases in seagrass shoot density over time in the restored seagrass meadows influenced water flow and caused a shift from an erosional to a depositional environment [18]. In addition, low seagrass densities such as those we observed in the 4-year treatment accelerated flow around individual shoots, created turbulence, and increased sediment suspension in a manner similar to that observed in areas without seagrass habitat [32,33]. This mechanism also can explain the lack of change in organic matter and carbon content with depth in 0- and 4-year treatments. Our results suggested that shoot densities in the 4-year treatments (average 123.2 shoots m^-2^) were insufficient to reduce resuspension and shallow mixing of sediment compared to bare sediments; that by 10 years after seeding (average 428.7 shoots m^-2^) the seagrass meadow stabilized and trapped sediment more effectively allowing for sediment accretion. These results were consistent with previous studies where a significant increase in sediment stabilization in dense seagrass meadows promoted sediment accumulation compared to unvegetated areas [17,18].

Sedimentation rates from measured cores do not take into account any mixing from organisms. However, as the sediment environment becomes anoxic [6], the abundance of bioturbating organisms decreases greatly [15,34]. In addition, nutrient and sedimentation profiles exhibited trends that were inconsistent with the homogenization of sediments by bioturbation; consistent trends would have been indicated by straight, vertical profiles (Figure 1, Figure 2). The 10-year treatment had a consistent accreting profile; bioturbation and roots most likely had little to no effect on the accretion profiles (Figure 2B). In addition, there was no evidence that roots impacted the ^210^Pb profiles because vertical profiles were observed only in the 0-year and 4-year treatments, but not in the 10-year treatment, which would have had more root activity and growth due to increases in seagrass densities (Figure 2A and 2B).

Sediment accretion rates and % C in the 10-year treatment showed a steady-state accretion rate before seeding, and then a significant increase in carbon burial rates 10 years after the seeding initiated seagrass meadow development (Figure 3A). However, following the seeding event, there was approximately a 5-year lag before there was a doubling in the carbon burial rate, compared to past trends. This can be attributed to changes in seagrass density at this site, where a large increase in seagrass density took approximately 4 years, which coincided with the dramatic increase in sediment accretion rates observed in the present study [3]. The reproductive phenology of *Zostera* *marina* in this region is such that seedlings typically flower and produce seeds in their second year; thereafter, those seeds that germinate and survive then produce seeds again after 2 years [26], resulting in an approximately 4-year lag in the rate of shoot density increase. Seed dispersal and shoot recruitment had a greater impact than clonal expansion for the meadow establishment, as evidenced by the large spatial scale of rapid meadow expansion [26]. Given the hydrological similarities between sites [3], we anticipate that the 4-year treatment will follow the same trajectory as the 10-year treatment and have similar accretion rates in the future as shoot densities increase rapidly. In addition, the % C data during this initial lag period suggests that the organic carbon source remained consistent, but that after the 5-year lag period there was a significant decrease in % C, indicating that the carbon accumulating in the sediment consisted of different sources of material. This suggests that initially during the lag period, low rates of carbon accumulation in seagrass sediments was most likely from seagrass detritus, and that once the restored seagrass meadows became more dense, there was increased trapping of particles such as seston and other allochthonous sources. By volume these seston particles would have lower % C compared to seagrass leaves but accumulate at a faster rate that corresponded to the increase in the carbon burial rate [20].

The 10-year restored seagrass meadows facilitated the accumulation of 36.68 (±2.79) g C m^-2^ yr^-1^, which falls just slightly below the range for carbon burial in natural seagrass meadows (45–190 g C m^-2^ yr^-1^) estimated by Mcleod et al. [7]. Because the restored seagrass in this area was still expanding and increasing in density at the time of this study, the carbon accumulation rate for the restored seagrass meadows will likely continue to increase. If we assume a linear increase in seagrass density based on current measurements and the annual carbon accumulation rate related to seagrass density [3], we can estimate the near-term carbon accumulation rates for these restored seagrass meadows. Applying the short-term linear trend to the last 5 years of carbon accumulation rates, we estimated a rate of 47.06 g C m^-2^ yr^-1^ by 12 years after seeding (2 years beyond the sampling reported here) that is within the measured range of natural seagrass meadows reported by Mcleod et al. [7].

Due to the inconsistencies of methods, there has been little consensus in past studies on including roots and rhizomes in sediment carbon measurements, which could potentially lead to higher estimates of carbon accumulation if roots were included [4]. To ensure that the removal of roots did not significantly influence bulk density or carbon content in the sediment, small subsets of cores were collected in summer of 2012, and we found no significant differences in bulk density within each age treatment with or without roots (T-test, 4-yr *p* = 0.581, *n* = 161; 10-yr *p* =0.570, *n* = 171). We also addressed this issue by analyzing the bulk carbon concentration for two additional cores (one from each of the 4-year and 10-year treatments) in which roots were not removed. We found that the average belowground biomass carbon stock was lower for the 4-year treatment (3.19 g C m^-2^, (*n* = 6, SE = 2.36) vs. 9.67 g C m^-2^ (*n* = 6, SE = 6.65) for the 10-year treatment), and bulk density was significantly lower with the presence of more roots in the 10-year treatment (Table 1). However, there was no significant difference for either the 4-year or 10-year treatment in bulk carbon measurements for the top 10 cm between cores with and without roots and rhizomes (X^2^ < 16.92; df = 9). This indicates that the roots and rhizome contribution to the carbon stock for these developing seagrass meadows were minimal compared to that of other particulate carbon in the sediments. Future studies should determine the relative contribution of seagrass root and rhizome carbon to estimated accumulation rates, as this may be important in older or more established meadows.

Seagrass ecosystems are lost each year through habitat destruction, eutrophication, and other anthropogenic stressors [8]. However, restoration, such as that occurring at the VCR LTER, can help mitigate the loss of habitat and associated ecosystem services [3,26]. Seagrass ecosystems have only recently received global recognition for their ability to sequester carbon [4,7], and carbon accumulation rates have only been measured for a few systems and species [6,11]. Until this study, there has not been any work on how or when restored seagrass systems promote the accumulation of carbon. Under current estimates of the economic cost of $41 per ton of CO_2_ [35] and 2011 estimates of restored seagrass coverage at the VCR LTER of 1700 ha [3], the restored seagrass provides an estimated social cost of approximately $7,000 yr^-1^ or $4.10 ha^-1^ yr^-1^ of carbon storage. These carbon accumulation rates will be useful for planners and policy makers in assessing the potential of restored seagrass ecosystems to sequester “blue carbon”.
